# Evaluation of Torsional Resistance and Bending Stiffness of Coronal Flaring Nickel-Titanium Instruments

**DOI:** 10.14744/eej.2021.25238

**Published:** 2021-11-18

**Authors:** Theodoro WEISSHEIMER, Murilo Priori ALCALDE, Julia Barrionuevo CORTEZ, Ricardo Abreu da ROSA, Rodrigo Ricci VIVAN, Pedro Henrique Souza CALEFI, Marco Antonio Hungaro DUARTE, Marcus Vinicius Reis SÓ

**Affiliations:** 1. Department of Conservative Dentistry, Federal University of Rio Grande do Sul, Porto Alegre, Brazil; 2. Health Science Center, Sacred Heart University, Bauru, SP, Brazil; 3. Department of Operative Dentistry, Endodontics and Dental Materials, Bauru School of Dentistry, University of São Paulo, Bauru, SP, Brazil

**Keywords:** Bending stiffness, coronal flaring, nickel-titanium, torsional resistance

## Abstract

**Objective:**

The aim of this study was to evaluate the bending and torsional resistance of the following instruments: Mtwo 25/.07 (MT - VDW, Munich, Germany), Navigator W-XN 25.07 (WXN - Wizard Navigator, Medin, Nové Město na Moravě, Czech Republic), ProTaper Universal SX 19/.04 (PSX - Dentsply Tulsa Dental Specialties, Tulsa, USA), MK Orifice Shapper 17/.08 (OS - MK Life Medical and Dental Products, Porto Alegre, Brazil) and MK Sequence 17.12 (MKS - MK Life Medical and Dental Products, Porto Alegre, Brazil).

**Methods:**

One hundred instruments were used (n=20). Resistance to bending (n=10), torque and angular deflection (n=10) at the failure of new instruments were measured according to ISO 3630-1. Metal mass volume at 3 mm from the tip was evaluated using micro-computed tomography (micro-CT). The fractured surface of each fragment was examined by scanning electron microscopy (SEM). Data were analysed using 1-way analysis of variance and Tukey tests.

**Results:**

Torsional resistance values of MK Sequence were higher than the other groups (P<0.05). No differences were observed among MT, WXN and OS (P>0.05) and PTS, which presented the lowest values (P<0.05). MT showed the highest angular deflection (P<0.05). WXN and PSX presented no significant difference (P>0.05). PSX and OS also showed no significant differences (P>0.05). MKS instruments had the lowest angular deflection values (P<0.05). There were significant differences among all the groups in bending stiffness test (P<0.05), but PSX had the lowest torque to bend (P<0.05). MKS had the larger metal mass volume at 3 mm from the tip (P<0.05). SEM analysis showed similar and typical features of torsional failure for all instruments tested.

**Conclusion:**

In conclusion, MK Sequence 17/.12 had the highest torsional fracture resistance. Mtwo 25/.07 showed higher angular deflection capacity, and ProTaper Universal SX the 19/.04 lower bending stiffness.

## Introduction

Nickel-titanium (NiTi) instruments significantly improved root canal preparation (1). The development of thermal treatments, different geometries, and kinematic changes have allowed more predictable root canal preparations (2). Coronal flaring NiTi instruments are usually presented in a shorter cutting length and a greater taper than NiTi instruments intended to shape the complete working length of the canal. Coronal flaring instruments are indicated before the negotiation of the middle and apical third to remove interferences from the root canal orifice (3), promoting early access of irrigants (4), facilitating the apical file size determination (5), reducing the apical extrusion of debris (6) and reducing the fracture incidence of the subsequent instruments (7, 8). Compared to stainless steel coronal flaring instruments, NiTi instruments are more flexible (9); however, they are still subject to failures that can lead to complications during treatment.

**Highlights**

• The characteristics of the instruments presented a great influence on their mechanical properties;
• Heat treatment was not a determining factor;
• MK Sequence 17/.12 had the highest torsional resistance, Mtwo 25/.07 the higher angular deflection and ProTaper Universal SX 19/.04 the lower bending stiffness.

Instrument fracture can occur through two different mechanisms. First, when the instrument repeatedly rotates in a curved canal, generating areas of tension and compression stress until flexural fatigue fracture occurs (10). Second, when the instrument binds to dentine, if excess torque onto the file, plastic deformation or a torsional fracture occurs (10, 11).

Among the many NiTi coronal flaring instruments available, ProTaper Universal SX (Dentsply Tulsa Dental Specialties, Tulsa, OK) is a 19 mm rotary instrument with a conventional NiTi alloy, presenting a tip size #19, a .04 mm/mm taper on its first 3 mm, and a triangular convex cross-section; Navigator W-XN 25/.07 (Wizard Navigator, Medin, NovéMěsto na Moravě, Czech Republic) is a 19 mm rotary instrument with a conventional NiTi alloy, that presents a tip size #25, a .07 mm/mm taper on its first 3 mm, and a triangular convex cross-section; MK Orifice Shapper 17/.08 (MK Life Medical and Dental Products, Porto Alegre, Brazil) is also a 19 mm rotary instrument with a conventional NiTi alloy, presenting a tip size #17, a .08 mm/mm taper on its first 3 mm, and a triangular convex cross-section; MK Sequence 17/.12 (MK Life Medical and Dental Products) is a 19 mm rotary instrument with a heat treatment similar to Blue treatment, presenting a tip size #19, a .12 mm/mm taper on its first 3 mm, and also a triangular convex cross-section; Mtwo 25/.07 (VDW, Munich, Germany) is a 25 mm rotary instrument presenting a conventional NiTi alloy, with a tip size #25, a .07 mm/mm taper on its first 3 mm, and a S-shaped cross-section.

These instruments are often used in narrow and constricted canals (8), therefore, understanding its mechanical properties can help the clinician in maintaining safety during clinical practice. So the aim of this study was to evaluate the bending stiffness and torsional (maximum torque load and angular deflection) resistance of 5 NiTi instruments used for coronal flaring and to obtain more knowledge about these instruments and to promote safer clinical practice. The null hypotheses tested were: (i) there are no differences in the torsional resistance among the tested instruments; (ii) there are no differences in the angular deflection values among the tested instruments; (iii) there are no differences in the bending stiffness among the files.

## Materials and Methods

Sample size calculation was performed before the mechanical testing using G*Power v.3.1 for Mac (Heinrich Heine, University of Düsseldorf, Düsseldorf, Germany) and by selecting the Wilcoxon–Mann-Whitney test. The alpha-type error of .05, beta power of .95, and N2/N1 ratio of 1 were also stipulated. The test calculated a total of 8 samples for each group as the ideal size for noting significant differences. However, an additional 20% of the total instruments was used to compensate for possible atypical values that might lead to sample loss.

A total of 100 NiTi instruments were used for this study. The samples were divided into five groups (n=20), as follows: Mtwo 25/.07 (MT), Navigator W-XN 25/.07 (WXN), ProTaper Universal SX 19/.04 (PSX), MK Sequence 17/.12 (MKS), and MK Orifice Shaper 17/.08 (OS). All files were inspected under a stereomicroscope (Carl Zeiss, LLC, EUA) at 16x magnification to detect possible defects or deformities previous to the tests.

### Torsional fatigue test

The torsional tests were performed based on the ISO 3630-1 (12), as previously reported (11). A total of 10 instruments were used in each group. Before testing, the handles of all instruments were removed at the point where they were attached to the torsion shaft. All MT instruments were cut at 19 mm from the tip, in order to reduce bias.

**Figure 1.**
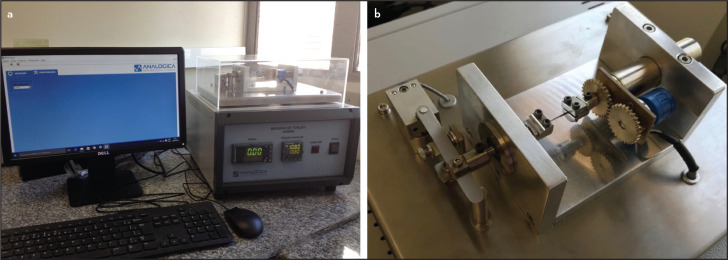
(a) Torsional testing machine and software. (b) Torsional testing apparatus, according to ISO 3630-1

The end of the shaft was clamped into a chuck connected to a geared motor of the torsion machine (Analógica, Belo Horizonte, Minas Gerais, Brazil). The first 3 mm of the instrument tips were clamped in another chuck. Rotation was started in the clockwise motion, at a speed of 2 rpm for all groups. The values of ultimate load and angular rotation were measured and controlled by a resistive angular transducer connected to a process controller, and recorded by a machine's specific program (MicroTorque; Analógica, Belo Horizonte, Minas Gerais, Brazil (Fig. 1).

### Bending stiffness test

The bending stiffness test was performed using the same torsion machine and program described for the torsional fatigue test. In addition, the methodology was adapted to the ISO 3630-1, as previously described (13).

A total of 10 instruments of each group were used to evaluate the flexibility and the maximum strength demanded to bend the files at 45° with its long axis. Again, all MT instruments were cut at 19 mm from the tip, in order to reduce bias.

Instruments were fixed at 3 mm from the tip perpendicular to the geared motor's axis. The flexural strength required to bend the instruments was automatically measured by the load cell and recorded.

### Metal mass volume analysis

A total of 10 instruments of each brand were scanned by micro-CT (Skycan 1174; Bruker-micro-CT, Kontich, Belgium) to evaluate the metal mass volume (mm^3^) along the 3 mm tip of all instruments, as previously reported (14, 15). The micro-CT parameters used were 50 kV, 800 mA, 360° of rotation, an isotropic resolution of 14.1 μm and a 0.5 mm-thick aluminium filter. The images were reconstructed with dedicated software (NRecon v. 1.6.3, Bruker-micro-CT), which enabled three-dimensional analyses. Therefore, the metal mass volume from the instrument tip until the third mm from this initial point of each instrument was measured using the CTan software v.1.12 (Bruker-micro-CT, Kontich, Belgium).

### Scanning electron microscopy evaluation

After the torsional test, all instruments were examined under scanning electron microscopy (SEM - JEOL, JSM-TLLOA, Tokyo, Japan) to determine the topographic features of the fragments. All instruments were cleaned in an ultrasonic cleaning device (Gnatus, Ribeirão Preto, São Paulo, Brazil) in distilled water for 3 minutes before SEM evaluation. The fractured surface of the instruments was examined at x200 and x1000 magnification in the centre of the surface.

### Statistical analysis

Shapiro-Wilk test was performed to verify the presence or absence of normality. The one-way analysis of variance (ANOVA) and Tukey tests were used for multiple and individual comparisons. The Prism 6.0 software (GraphPad Software Inc., La Jolla, CA, USA) was used as the analytical tool, and the level of significance was set at 5%.

## Results

Table 1 presents the mean and standard deviations of torsional fatigue (torque maximum torsional strength and angular deflection) and bending stiffness for each instrument. MKS instruments had significantly higher torsional strength values than all other instruments (P<0.05). There was no difference among MT, WXN and OS (P>0.05). PSX instruments had the lowest torsional strength (P<0.05).

**Table 1.** Mean values and standard deviation (SD) of Torque (Ncm), angular deflection (°) and Torque (Ncm) at bending moment of instruments tested

Instruments	Torque	Angular Deflection	Bending
	Mean	SD	Mean	SD	Mean	SD
MT 25/.07	1.13^a^	0.15	440.8^a^	44.35	0.57^a^	0.05
WXN 25/.07	1.11^a^	0.13	387.6^b^	22.23	0.81^b^	0.07
PSX 19/.04	0.57^b^	0.06	365.1^b,c^	22.43	0.43^c^	0.04
MKS 17/.12	1.69^c^	0.06	305.2^d^	7.28	1.56^d^	0.06
OS 17/.08	1.19^a^	0.12	343.7^c^	45.06	0.73^e^	0.07

Different superscript letters in the same column indicate statistical differences among groups (P<0.05). Ncm: Newtons centimeter, MT: Mtwo, WXN: Navigator W-XN, PSX: ProTaper Universal SX, MKS: MK Sequence, OS: MK Orifice Shaper

After the angular deflection assessment, MT had the highest values compared with all the groups (P<0.05). There was no difference between WXN and PSX (P>0.05), and between PSX and OS (P>0.05). The MKS instruments had the lowest angular deflection values (P<0.05).

The bending test showed that there were differences among all the instruments (P<0.05). MKS instruments had the highest strength to bend, followed by WXN, OS, and MT. PSX instruments had the lowest torque to bend than all the groups (P<0.05).

Table 2 presents the metal mass volume analysis on the first 3 mm of the tip of the instruments. There were differences among all the files (P<0.05). MKS instruments had the highest metal mass volume, followed by OS, WXN, and MT instruments. PSX instruments had the lowest metal mass volume of all the groups (P<0.05).

**Table 2.** Mean and standard deviation (SD) values of metal mass volume (mm^3^) 3 mm from the instrument’s tip

Instruments	Volume of metal mass (mm^3^)
	Mean	SD
MT 25/.07	0.095^b^	0.0010
WXN 27/.07	0.110^c^	0.0070
PSX 19/.04	0.055^d^	0.0038
MKS 17/.12	0.185^a^	0.0063
OS 17/.08	0.145^e^	0.0051

Different superscript letters in the same column indicate statistical differences among groups (P<0.05). MT: Mtwo, WXN: Navigator W-XN, PSX: ProTaper Universal SX, MKS: MK Sequence, OS: MK Orifice Shaper

**Figure 2.**
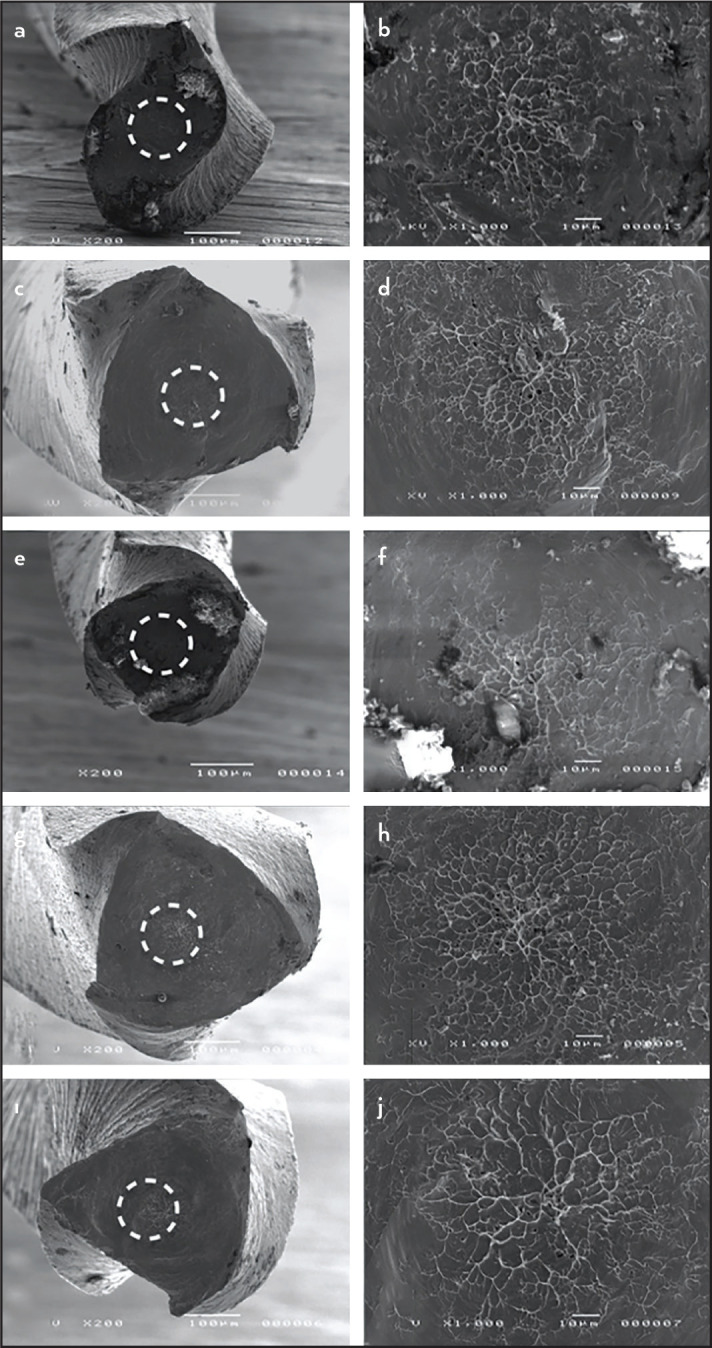
Scanning electron microscopy images of the fractured surfaces of Mtwo 25/.07 (a, b), W-XN (c, d), SX (e, f), Sequence 17/.12 (g, h) and Orifice Shaper (ı, j) after torsional testing. The left column shows images with the circular boxes indicating concentric abrasion marks at 200x magnification; the right column shows concentric abrasion marks at 1000x magnification. The skewed dimples near the centre of rotation are typical features of torsional failure

SEM analysis revealed that all instruments showed abrasion marks and fibrous dimples near the centre of rotation for torsional fracture (Fig. 2).

## Discussion

NiTi coronal flaring instruments are often used to promote the enlargement of the entrance of narrow and constricted canals. Thus, the instrument's tip is usually subjected to greater stress, imposing a greater risk of torsional fracture (16). This occurrence can compromise the endodontic procedure and, for this reason, the study of its mechanical properties is essential.

Regarding torsional resistance, the null hypothesis was rejected. The torsional test showed that MKS instruments had the highest torsional strength. All instruments of this study have triangular cross-section, except MT that has S-shaped design. An evaluation of the instrument's metal mass volume at 3mm from the tip was performed using micro-CT. PSX presented the smallest metal mass volume (0.055 mm^3^), followed by MT (0.095 mm^3^), WXN (0.110mm^3^) and OS instruments (0.145 mm^3^). Finally, the larger metal mass volume was verified in MKS instruments (0.185 mm^3^).

Some authors suggested that the torsional properties of NiTi instruments could be affected by instruments features (e.g. taper and cross-section), which can affect the metal mass volume, increasing its torsional resistance (14, 15, 17-20). On the other hand, a recent study demonstrated that instruments with equal cross-section and metal mass volume can still present different torsional resistance due to the instruments' polar moment of inertia (21). These differences among different cross-sections, metal mass volume and even polar moment of inertia probably justify the results presented by the tested instruments.

Although MKS instruments has a heat-treated alloy, a previous study that verified the properties of coronal flaring instruments, with and without heat-treatment concluded that this is not a factor that influences the torsional resistance for these instruments (22). Heat treatment promotes greater flexibility and angular deflection capacity to instruments (20, 23). However, instruments specifically designed for cervical preparation have geometric features (taper and cross-sections) that provide greater metal mass volume and stiffness. Therefore, although some instruments have heat-treated NiTi alloys, the design probably modified their mechanical properties, explaining the results of this study. According to the manufacturer, MKS instruments possess a heat treatment similar to the Blue treatment, which should provide greater flexibility. However, this instrument demonstrated greater torque resistance, less angular deflection and greater bending stiffness, showing that the design of the instrument can significantly impact their mechanical properties. In addition, the other instruments tested in this study are made with conventional NiTi alloy and presented different results so that these differences can be related to the geometrical features.

When evaluating the findings on angular deflection, the second null hypothesis was rejected. In this study, the highest angular deflection values were presented by MT instruments. High values of angular deflection mean considerable strain before the moment of failure, functioning as a safety factor in clinical practice (24).

Although MT instruments presents a conventional NiTi alloy, this result may be related to the instrument S-shaped cross-section and small metal mass volume, favouring a greater deformation previous to failure (17, 18). On the other hand, the lowest values presented by MKS can be explained due to these instruments' larger metal mass volume. Previous studies (17, 25) had reported that instruments with larger metal mass volume tend to present flexibility and lower angular deflection to fracture, which could explain the results of this study.

Recent studies have shown that instruments with longer length tend to present greater torque resistance compared to instruments of shorter length (26, 27). On the other hand, angular deflection does not appear to be influenced (27). Therefore, it is important to note that although the length standardization was carried out with a methodological purpose, we cannot affirm that the MT instruments with 25 mm length present the same results in clinical situations as presented in this study, being a limitation of this study.

As for the results on the bending stiffness, the third null hypothesis was also rejected. Bending stiffness is related to instrument performance when used in curved canals (28). However, in coronal flaring instruments, this property is related to performing brushing movements against the dentinal projections (22).

MKS instruments needed the highest strength to bend (P<0.05), and PSX the lowest. Although a previous study has shown that coronal flaring heat-treated instruments presented lower bending stiffness when compared to the same file without heat treatment (4), our results were different mainly because of the larger taper of the heat-treated instruments used in this study. MKS and PSX instruments present both triangular convex cross-sections. However, although PSX presents a greater tip when compared to MKS, the second presents a greater taper increase, thus a larger metal mass volume, explaining the results of this study.

As for the implications of this study for clinical practice, the highest torsional strength and bending stiffness of MKS instruments indicates that these instruments would probably suffer less risks regarding torsional fracture in narrow and constricted canals and would be able to remove dentin projections more efficiently. On the other hand, the greater angular deflection presented by MT instruments could benefit clinicians, indicating plastic/permanent deformation and imminent fracture before it occurred (24).

Although some mechanical properties of these instruments have been evaluated, it is not possible to compare their performance and clinical safety by this study. Therefore, the authors suggest that future studies should be carried out to evaluate the cutting efficiency and its effectiveness in the cervical preparation of the root canals, which may complement our results.

## Conclusion

Within the limitations of this study, the characteristics of the instruments, such as cross-sectional designs, tip size and taper, had a great influence on the mechanical properties of the instruments tested. Heat treatment was not a determining factor. Our results showed that MK Sequence 17/.12 obtained the highest torsional fracture resistance when compared to other instruments. In addition, Mtwo 25/.07 presented higher angular deflection capacity and ProTaper Universal SX 19/.04 the lower bending stiffness.

### Disclosures

**Conflict of interest:** The authors deny any conflict of interest.

**Ethics Committee Approval:** This study did not need ethics committee approval as it does not involve human or animal material.

**Peer-review:** Externally peer-reviewed.

**Financial Disclosure:** This study was supported by the State of São Paulo Research Foundation (FAPESP 2018/23452-8).

**Authorship contributions:** Concept – M.V.R.S., M.P.A., R.R.V., R.A.D.R., M.A.H.D.; Design – M.V.R.S., M.P.A., R.R.V., R.A.D.R., M.A.H.D.; Supervision – M.P.A.; Funding - M.P.A., J.B.C.; Materials - None; Data collection &/or processing – M.P.A., J.B.C., P.H.S.C.; Analysis and/or interpretation – M.V.R.S., M.P.A., R.R.V., R.A.D.R., M.A.H.D., T.W.; Literature search – T.W., M.P.A., M.V.R.S.; Writing – T.W., M.P.A.; Critical Review – M.V.R.S., R.A.D.R., R.R.V., M.A.H.D.
